# Long COVID and Food Insecurity in US Adults, 2022-2023

**DOI:** 10.1001/jamanetworkopen.2025.30730

**Published:** 2025-09-09

**Authors:** John C. Lin, Madison McCarthy, Sriya Potluri, Dang Nguyen, Ruiqi Yan, Jaya Aysola

**Affiliations:** 1 Department of Medicine, Perelman School of Medicine, University of Pennsylvania, Philadelphia; 2 Penn Medicine Center for Health Equity Advancement, University of Pennsylvania Health System, Philadelphia; 3 Yale School of Public Health, New Haven, Connecticut; 4 University of Cambridge, Cambridge, United Kingdom; 5 Harvard T.H. Chan School of Public Health, Harvard University, Boston, Massachusetts; 6 Department of Pediatrics, Perelman School of Medicine, University of Pennsylvania, Philadelphia

## Abstract

**Importance:**

Long COVID (ie, post–COVID-19 condition) is a substantial public health concern, and its association with health-related social needs, such as food insecurity, remains poorly understood. Identifying modifiable risk factors like food insecurity and interventions like food assistance programs is critical for reducing the health burden of long COVID.

**Objective:**

To investigate the association of food insecurity with long COVID and to assess the modifying factors of Supplemental Nutrition Assistance Program (SNAP) participation and employment status.

**Design, Setting, and Participants:**

This retrospective, cross-sectional survey study used data from the 2022 to 2023 National Health Interview Survey. Respondents aged 18 years and older who reported prior COVID-19 infection and responded to questions on food insecurity and long COVID were included.

**Exposure:**

Food insecurity, categorized as food secure or food insecure.

**Main Outcomes and Measures:**

The primary outcome was current long COVID, defined as symptoms lasting 3 or more months after initial COVID-19 infection persisting to time of interview. The secondary outcome was long COVID recovery, indicating history of long COVID without current symptoms. Food insecurity was measured using the validated 10-item National Center for Health Statistics food insecurity scale. Odds ratios (ORs) and 95% CIs for the association of food insecurity with long COVID were calculated using simple and multiple logistic regression.

**Results:**

The study enrolled 21 631 participants (1255 female [weighted percentage, 53%]; 5058 aged 65 years or older [weighted percentage, 16%]), including 19 824 with food security and 1807 with food insecurity. In total, 288 respondents with food insecurity (weighted percentage, 15%) reported current long COVID compared with 1547 (weighted percentage, 7%) without food insecurity. Food insecurity was positively associated with current long COVID (adjusted OR, 1.73; 95% CI, 1.39-2.15) and negatively associated with recovery among adults with prior long COVID (adjusted OR, 0.70; 95% CI, 0.54-0.92). SNAP participation (*P *for interaction = .04) and unemployment (*P *for interaction = .04) significantly modified these associations.

**Conclusions and Relevance:**

In this survey study of US adults with prior COVID-19 infection, food insecurity was associated with greater odds of long COVID and lower odds of recovery, with SNAP participation and unemployment mitigating these associations. These findings suggest that expanding SNAP eligibility, simplifying enrollment processes, and increasing awareness of food assistance programs may reduce the burden of food insecurity and long COVID and further emphasize the importance of addressing health-related social needs in chronic disease prevention and management.

## Key Points

**Question:**

Is food insecurity associated with increased risk of long COVID (ie, post–COVID-19 condition) and lower odds of recovery among US adults with prior COVID-19 infection?

**Findings:**

In this survey study of 21 631 US adults who previously had COVID-19, those who experienced food insecurity were more likely to report post–COVID-19 condition. Specifically, food insecurity was associated with a 73% higher chance of having current long COVID and a 30% lower chance of recovering from it; however, participation in food assistance programs like the Supplemental Nutrition Assistance Program (SNAP) and being employed appeared to reduce these risks.

**Meaning:**

These findings suggest that food insecurity may be an important and modifiable risk factor for long COVID and that strengthening access to programs like SNAP, raising awareness, and simplifying enrollment could help reduce the health burden of long COVID.

## Teaser

This survey study analyzes associations of post–COVID-19 condition (ie, long COVID) with food insecurity among US adults based on the 2022 to 2023 National Health Interview Survey.

## Introduction

Persistent symptoms beyond 3 months following COVID-19 infection, known as long COVID or post–COVID-19 condition, is considered a chronic condition of substantial public health concern.^1^ Long COVID occurs in more than 10% of severe COVID-19 infections, impacting an estimated 65 million individuals worldwide.^2^ Although much research has focused on the clinical presentation and indications of long COVID, less attention has been given to the broader social and economic impacts of having it.^3^ Emerging evidence suggests that individuals with long COVID may be at higher risk for unemployment and health care–related financial toxicity.^4,5^ Conversely, there is also evidence to suggest that individuals with lower income are at greater risk for long COVID.^6^ While income has been identified as a potential contributing factor to Long COVID, we lack knowledge about the role of other potential social risk factors, such as food insecurity.

Prior work has found a large-magnitude association of food insecurity with delayed or forgone medical care, worsened mental health, and racial disparities during the COVID-19 pandemic.^7,8,9^ Moreover, food insecurity, particularly, has been associated with increased risk for well-defined chronic health conditions, such as heart disease and diabetes.^10^ However, the association of long COVID with key health-related social needs such as food insecurity remains unexplored.

To our knowledge, no prior work has examined the association of food insecurity with long COVID. Understanding this association is critical given the prevalence of food insecurity in the US and the scale of federal food assistance programs. Approximately 13.5% of US households were food insecure in 2023.^11^ Although unemployment is associated with increased food insecurity,^11^ unemployment benefits were associated with decreased food insecurity during the COVID-19 pandemic.^12,13^ The Supplemental Nutrition Assistance Program (SNAP) is the largest government-funded food assistance program to combat food insecurity in the US, with a monthly average of 42.1 million participants in 2023.^14^ SNAP participation is associated with a reduction in food insecurity at the individual and household levels.^15^ In this study, we investigate the association of food insecurity with long COVID using 2022 to 2023 National Health Interview Survey (NHIS) data and analyze the interactions of SNAP and unemployment with this association.

## Methods

### Data Source

This survey study was determined exempt from institutional review board approval and the requirement of informed consent because the NHIS is a publicly available, deidentified data set. Reporting followed the American Association for Public Opinion Research (AAPOR) reporting guideline.^16^ The NHIS is an annual, nationally representative, cross-sectional survey conducted by the National Center for Health Statistics (NCHS).^8^ It collects a wide range of demographic, socioeconomic, and health-related information from the civilian, noninstitutionalized population of the US. Response rates for 2022 and 2023 data were 47.7% and 47.0%, respectively.^17^ Data from 2020 and 2021 were excluded because key questions on long COVID were not yet included. The NHIS methodology is detailed by the NCHS.^17^

### Study Population

For this study, we included individuals aged 18 years and older from the NHIS adult sample who reported having COVID-19 at any point and provided responses to questions regarding food insecurity. We excluded those respondents with missing data for the long COVID variable (139 individuals) or food insecurity variable (1267 individuals). For secondary analyses of long COVID recovery, we further restricted the sample to respondents who reported experiencing long COVID at any point.

### Outcome Variables

We examined associations with 2 binary outcome variables: (1) whether individuals had current long COVID (yes or no) and (2) whether individuals had a history of and recovered from long COVID (yes or no). Consistent with standard definitions,^18^ we defined long COVID as self-reported symptoms persisting for more than 3 months after initial COVID-19 diagnosis.^17^ We defined current long COVID as meeting the definition of long COVID plus the presence of these symptoms at time of interview.^17^ We categorized respondents as having recovered from long COVID if they reported ever having long COVID but not having these symptoms at time of interview.^17^

### Exposures of Interest

The NHIS questionnaire measured food insecurity using the NCHS-validated 10-item food insecurity scale, with scores of 0 to 2 indicating high or marginal food security and scores of 3 to 10 indicating low or very low food security.^19^ This measure was a validated and abbreviated version of the US Department of Agriculture Adult Food Security Survey Module with a 30-day reference period.^19,20^ Additional information about this measure can be found in NCHS methodology report as well as prior studies using this measure.^17,19^

### Additional Covariates

Additional covariates included demographics such as age, sex, and race and ethnicity, as well as household income relative to the federal poverty level (FPL), education, geographic region, employment history in the past week, weight status (defined by body mass index), smoking, health insurance (private, Medicare [including dual eligibles], Medicaid and other public, other coverage, and uninsured or self-pay), number of COVID-19 vaccinations (0, 1-2, and ≥3), and number of other chronic conditions (arthritis, asthma, cancer, chronic obstructive pulmonary disease, diabetes, and hypertension). Self-reported race and ethnicity included the following categories: American Indian and Alaska Native, Hispanic, non-Hispanic Asian, non-Hispanic Black, non-Hispanic White, and other (defined as multiracial individuals and people whose race group was not releasable for respondent confidentiality); race and ethnicity were included to account for documented racial and ethnic disparities in both food insecurity and long COVID prevalence that may reflect underlying structural and social determinants of health. Chronic conditions were included based on their prevalence and usage in prior NHIS studies.^21,22^ We also recorded household participation in the SNAP in the past month.

### Missing Data

We excluded respondents with missing data for long COVID and food insecurity. For all categorical covariates unrelated to our exposures and outcomes of interest, missing values were replaced with a distinct placeholder value (9999) that did not overlap with any observed categories in all regression models to account for potential bias due to missingness. Number of other chronic conditions, the only ordinal variable, had 6 missing values, which were dropped from analysis in multiple regression. We excluded persons with a survey response of unknown COVID-19 vaccination status (26% of the sample) from the main regression analyses to ensure interpretability and reduce misclassification bias. Given the percentage of missingness of this variable, we included those that reported unknown for COVID-19 vaccination as a separate category in sensitivity analyses to examine model associations.

### Statistical Analysis

First, we characterized the prevalence of food insecurity among adults with and without long COVID. We compared people with and without current long COVID using Rao-Scott χ^2^ tests for categorical variables. We evaluated the association of food insecurity with current long COVID using simple and multiple logistic regression, adjusting for age, race and ethnicity, sex, income, region, education, employment, insurance, weight status, smoking history, COVID-19 vaccinations, and chronic conditions. We further examined the association of food insecurity with long COVID recovery using similar logistic regression models. All models on recovery were restricted to people with long COVID, comparing individuals who had recovered from long COVID with those with ongoing symptoms. To assess a potential dose-response association of vaccination with long COVID, we modeled vaccination status as a categorical variable (0, 1-2, or ≥3 doses) and examined the direction and magnitude of regression coefficients. We also assessed linearity by plotting residuals across levels of vaccination status.

We then conducted a series of subgroup analyses to examine the interactions of SNAP participation and employment status on the association of food insecurity with long COVID, given the influence of SNAP participation and unemployment on food insecurity, particularly during the COVID-19 pandemic.^13,23,24^ We analyzed associations of food insecurity with long COVID among SNAP participants and nonparticipants and among employed and unemployed individuals. We also conducted subgroup analysis among people with incomes at 200% or less or greater than 200% FPL to approximate SNAP eligibility thresholds. We further assessed potential variations in these associations using interaction terms between food insecurity and SNAP participation, as well as food insecurity and employment status. We estimated odds ratios (ORs) and 95% CIs for interaction terms using the delta method, with margins and standard errors derived from the fitted logistic regression model by holding all other covariates at their mean values.

We conducted a sensitivity analysis using Poisson regression with robust standard errors given the risk of overestimation for common outcomes with logistic regression. We also conducted a sensitivity analysis to include those with unknown vaccination status due to the preventive effects of COVID-19 vaccination on long COVID .^25^ We applied NCHS sampling weights to account for the complex survey design and ensure national representativeness. No observations were removed in a manner that would disrupt the weighting structure. We used Stata Version 18.0 (StataCorp) to perform all statistical analyses. Statistical significance was defined as a 2-sided *P* < .05.

## Results

In total, this study included 21 631 adults (19 824 with food security and 1807 with food insecurity), and this surveyed population was nationally representative of approximately 205 million adults after weighting. Demographics and baseline characteristics of people with and without current long COVID are described in Table 1. Of the estimated nationally representative population, 12 155 (weighted percentage, 53%) were female, 3578 (weighted percentage, 19%) were Hispanic, 1906 (weighted percentage, 9%) were non-Hispanic Black, 14 456 (weighted percentage, 64%) were non-Hispanic White, and 5058 (weighted percentage, 16%) were 65 years or older. In total, 288 respondents (weighted percentage, 15%) of those with food insecurity reported current long COVID compared with 1547 respondents (weighted percentage, 7%) without food insecurity. Additionally, 5540 respondents (weighted percentage, 26%) declined to describe their COVID-19 vaccination status.

**Table 1.** Comparison of Respondent Characteristics by Food Insecurity Status^a^

Characteristic	Participants, No. (weighted %)			*P* value
	Overall (N = 21 631)	Food secure (n = 19 824)	Food insecure (n = 1807)	
Age, y				
18-30	3802 (25)	3418 (24)	384 (27)	<.001
31-50	7693 (37)	6914 (37)	779 (43)	
51-64	5052 (22)	4655 (22)	397 (20)	
≥65	5058 (16)	4814 (17)	244 (9)	
Unknown or missing	26 (<1)	23 (<1)	3 (<1)	
Sex				
Female	12 155 (53)	10 946 (52)	1209 (64)	<.001
Male	9473 (47)	8875 (48)	598 (36)	
Unknown or missing	3 (<1)	3 (<1)	0	
Race and ethnicity				
American Indian and Alaska Native	316 (1)	253 (1)	63 (3)	<.001
Hispanic	3578 (19)	3097 (18)	481 (29)	
Non-Hispanic Asian	1124 (5)	1085 (6)	39 (2)	
Non-Hispanic Black	1906 (9)	1579 (9)	327 (18)	
Non-Hispanic White	14 456 (64)	13 588 (66)	868 (46)	
Other^b^	251 (1)	222 (1)	29 (2)	
Household income				
<100% FPL	1829 (8)	1301 (6)	528 (28)	<.001
100%-199% FPL	3403 (16)	2751 (14)	652 (36)	
200%-399% FPL	6354 (30)	5874 (30)	480 (28)	
≥400% FPL	10 045 (46)	9898 (50)	147 (8)	
Education				
High school or less	6383 (33)	5523 (31)	860 (51)	.001
Some college	11 652 (53)	10 794 (54)	858 (45)	
Master’s or more	3516 (14)	3440 (15)	76 (3)	
Unknown or missing	80 (<1)	67 (<1)	13 (<1)	
Employment				
Yes	13 944 (68)	12 963 (69)	981 (56)	<.001
No	7651 (32)	6830 (31)	821 (44)	
Unknown or missing	36 (<1)	31 (<1)	5 (<1)	
Health insurance				
Private	14 177 (67)	13 486 (70)	691 (38)	<.001
Medicare^c^	2703 (9)	2520 (9)	183 (7)	
Medicaid and other public	2186 (12)	1605 (10)	581 (34)	
Other coverage	1103 (4)	995 (4)	108 (5)	
Uninsured	1401 (8)	1162 (7)	239 (15)	
Unknown or missing	61 (<1)	56 (<1)	5 (<1)	
US region				
Northeast	3500 (18)	3267 (18)	233 (14)	<.001
Midwest	4752 (21)	4386 (21)	366 (21)	
South	7932 (38)	7172 (37)	760 (41)	
West	5447 (24)	4999 (24)	448 (24)	
Smoking				
Never	14 201 (68)	13 208 (69)	993 (57)	<.001
Former	5440 (23)	5031 (23)	409 (21)	
Current	1947 (9)	1547 (8)	400 (21)	
Unknown or missing	43 (<1)	38 (<1)	5 (<1)	
Body mass index^d^				
Nonoverweight (<25.0)	6514 (30)	6060 (31)	454 (2)	<.001
Overweight (25.0-29.9)	7276 (33)	6792 (34)	484 (27)	
Obesity (≥30)	7455 (35)	6623 (3)	832 (46)	
Unknown or missing	386 (2)	349 (2)	37 (2)	
No. of COVID-19 vaccinations				
0	4108 (21)	3577 (20)	531 (30)	<.001
1-2	4724 (23)	4251 (22)	473 (26)	
≥3	7259 (30)	6867 (31)	392 (20)	
Unknown or missing	5540 (26)	5129 (27)	411 (24)	
No. of chronic conditions^e^				
0	9418 (48)	8795 (49)	623 (37)	<.001
1	6287 (29)	5780 (29)	507 (29)	
2	3464 (14)	3135 (14)	329 (18)	
3	1700 (6)	1487 (6)	213 (10)	
4	592 (2)	493 (2)	99 (5)	
5	142 (<1)	109 (<1)	33 (1)	
6	22 (<1)	19 (<1)	3 (<1)	
Unknown or missing	6 (<1)	6 (<1)	0	

Abbreviation: FPL, federal poverty level.

^a^ Sample weights were applied to all analyses to account for nonresponse bias and produce nationally representative population estimates. Participants without data on long COVID and food insecurity were excluded; for other variables, missing values were replaced with a distinct placeholder value (9999).

^b^ Other included multiracial individuals and people whose race group was not releasable for respondent confidentiality.

^c^ Respondents classified as having Medicare included those who were dual eligible for Medicaid and Medicare Advantage patients.

^d^ Calculated as weight in kilograms divided by height in meters squared.

^e^ Chronic conditions included arthritis, asthma, cancer, chronic obstructive pulmonary disease, diabetes, and hypertension.

Of those with food insecurity, 567 (weighted percentage, 32%) participated in SNAP, including 319 (weighted percentage, 60%) below 100% FPL; most people who were food insecure and did not participate in SNAP were in households at 100% to 199% FPL (465 individuals [weighted percentage, 37%]) or 200% or greater FPL (562 individuals [weighted percentage, 46%]). Most adults receiving SNAP (456 individuals [weighted percentage, 60%]) were not employed, compared with 456 individuals (weighted percentage, 37%) not receiving SNAP.

In simple regression, food insecurity showed a large positive association with current long COVID (OR, 2.46; 95% CI, 2.05-2.96). Logistic regression results for current long COVID are presented in Table 2. After adjusting for covariates in multiple regression, the association remained significant (adjusted OR [aOR], 1.73; 95% CI, 1.39-2.15). Other factors significantly associated with greater adjusted odds of long COVID included age (31-50 years: aOR, 1.64; 95% CI, 1.26-2.13; 51-64 years: aOR, 1.52; 95% CI, 1.16-1.99) relative to ages 18 to 30 years, Western region (aOR, 1.40; 95% CI, 1.08-1.82) vs Northeast, unemployment (aOR, 1.33; 95% CI, 1.11-1.60) vs employment, former smoking (aOR, 1.23; 95% CI, 1.03-1.46) or current smoking (aOR, 1.28; 95% CI, 1.00-1.62) vs never smoking, obesity (aOR, 1.23; 95% CI, 1.02-1.49) vs nonoverweight body mass index, and number of chronic conditions (aOR, 1.43; 95% CI, 1.34-1.53). Conversely, respondents who identified as males as compared with females were significantly less likely to report having long COVID (aOR, 0.58; 95% CI, 0.50-0.67). Similarly, those respondents who identified as non-Hispanic Black, as compared with non-Hispanic White, had a lower adjusted odds of reporting long COVID (aOR, 0.66; 95% CI, 0.50-0.86). Lastly, the number of COVID-19 vaccinations received was associated with lower odds of having long COVID in a dose response. Compared with individuals with 3 or more vaccine doses, those with 1 to 2 doses (β = 0.23; 95% CI, 0.38-0.76) and 0 doses (β = 0.57; 95% CI, 0.05-0.40) had higher adjusted odds of long COVID, demonstrating a monotonic dose-response association. Residual plots across vaccination levels showed no meaningful deviation from 0, supporting the linearity assumption.

**Table 2.** Unadjusted and Adjusted Odds of Current Long COVID by Food Insecurity Status and Other Respondent Characteristics

Characteristics	Current long COVID, OR (95% CI) (n = 16 048)	
	Unadjusted	Adjusted^a^
Food insecurity status		
Food secure	1 [Reference]	1 [Reference]
Food insecure	2.46 (2.05-2.96)^b^	1.73 (1.39-2.15)^b^
Age, y		
18-30	1 [Reference]	1 [Reference]
31-50	1.85 (1.43-2.40)^b^	1.64 (1.26-2.13)^b^
51-64	2.14 (1.67-2.73)^b^	1.52 (1.16-1.99)^b^
≥65	1.97 (1.53-2.53)^b^	1.05 (0.74-1.49)
Sex		
Female	1 [Reference]	1 [Reference]
Male	0.55 (0.48-0.63)^b^	0.58 (0.50-0.67)^b^
Race and ethnicity		
American Indian and Alaska Native	1.34 (0.87-2.05)	0.97 (0.61-1.55)
Hispanic	0.84 (0.68-1.05)	0.90 (0.72-1.13)
Non-Hispanic Asian	0.45 (0.28-0.72)^b^	0.65 (0.39-1.07)
Non-Hispanic Black	0.78 (0.60-1.01)	0.66 (0.50-0.86)^b^
Non-Hispanic White	1 [Reference]	1 [Reference]
Other^c^	0.61 (0.28-1.35)	0.68 (0.31-1.47)
Household income		
≥400% FPL	1 [Reference]	1 [Reference]
200%-399% FPL	1.31 (1.10-1.56)^b^	0.92 (0.68-1.23)
100%-199% FPL	1.79 (1.47-2.18)^b^	1.13 (0.89-1.44)
<100% FPL	1.80 (1.42-2.27)^b^	1.05 (0.87-1.27)
US region		
Northeast	1 [Reference]	1 [Reference]
Midwest	1.28 (1.00-1.64)	1.09 (0.84-1.41)
South	1.34 (1.06-1.70)^b^	1.18 (0.92-1.51)
West	1.38 (1.06-1.80)^b^	1.40 (1.08-1.82)^b^
Education		
Master’s or more	1 [Reference]	1 [Reference]
Some college	1.38 (1.12-1.71)^b^	1.04 (0.83-1.29)
High school or less	1.51 (1.19-1.90)^b^	0.91 (0.70-1.19)
Employment		
Yes	1 [Reference]	1 [Reference]
No	1.80 (1.57-2.07)^b^	1.33 (1.11-1.60)^b^
Insurance^d^		
Private only	1 [Reference]	1 [Reference]
Medicare	1.44 (1.18-1.77)^b^	1.06 (0.80-1.42)
Medicaid or other public	1.86 (1.52-2.26)^b^	1.17 (0.91-1.49)
Other coverage	1.87 (1.45-2.43)^b^	1.26 (0.94-1.69)
Uninsured	1.04 (0.78-1.39)	0.93 (0.68-1.28)
Smoking		
Never	1 [Reference]	1 [Reference]
Former	1.56 (1.33-1.83)^b^	1.23 (1.03-1.46)^b^
Current	1.92 (1.55-2.37)^b^	1.28 (1.00-1.62)^b^
Body mass index^e^		
Nonoverweight (<25.0)	1 [Reference]	1 [Reference]
Overweight (25.0-29.9)	1.12 (0.94-1.34)	1.05 (0.87-1.27)
Obesity (≥30)	1.64 (1.38-1.96)^b^	1.23 (1.02-1.49)^b^
No. of COVID-19 vaccinations		
≥3	1 [Reference]	1 [Reference]
1-2	1.13 (0.96-1.33)	1.25 (1.05-1.49)^b^
0	1.67 (1.41-1.98)^b^	1.76 (1.45-2.14)^b^
No. of chronic conditions	1.53 (1.45-1.60)^b^	1.43 (1.34-1.53)^b^

Abbreviations: FPL, federal poverty level; OR, odds ratio.

^a^ In multiple logistic regression, adjusted ORs and 95% CIs were calculated for current long COVID after adjusting for age, sex, race and ethnicity, household income, region, education, employment, insurance, smoking history, weight status, number of COVID-19 vaccinations, and other chronic conditions (arthritis, asthma, cancer, chronic obstructive pulmonary disease, diabetes, and hypertension). Sample weights were applied to all analyses to account for nonresponse bias and produce nationally representative population estimates. For categorical variables, missing values were replaced with a distinct placeholder value (9999). For number of chronic conditions (ordinal variable) and COVID-19 vaccination status, missing values were dropped.

^b^ Statistical significance (*P* < .05).

^c^ Other included multiracial individuals and people whose race group was not releasable for respondent confidentiality.

^d^ Respondents classified as having Medicare included those who were dual eligible for Medicaid and Medicare Advantage patients.

^e^ Calculated as weight in kilograms divided by height in meters squared.

Food insecurity was negatively associated with recovering from long COVID in simple (OR, 0.67; 95% CI, 0.53-0.86) and multiple regression (aOR, 0.70; 95% CI, 0.54-0.92) (Table 3). Older age (31-50 years: aOR, 0.66; 95% CI, 0.48-0.91; 51-64 years: aOR, 0.55; 95% CI, 0.47-0.91), unemployment (aOR, 0.67; 95% CI, 0.53-0.84), and number of chronic conditions (aOR, 0.82; 95% CI, 0.75-0.89) were inversely associated with long COVID recovery. Male relative to female sex (aOR, 1.25; 95% CI, 1.02-1.54) as well as non-Hispanic Black race (aOR, 1.51; 95% CI, 1.07-2.14) and other race and ethnicity (aOR, 2.44; 95% CI, 1.03-5.79) relative to non-Hispanic White race were positively associated with long COVID recovery. See Table 2 for a complete list of associations.

**Table 3.** Unadjusted and Adjusted Odds of Long COVID Recovery by Food Insecurity Status and Other Respondent Characteristics

Characteristics	Long COVID recovery, OR (95% CI) (n = 3877)	
	Unadjusted	Adjusted^a^
Food insecurity status		
No food insecurity	1 [Reference]	1 [Reference]
Food insecurity	0.67 (0.53-0.86)^b^	0.70 (0.54-0.92)^b^
Age, y		
18-30	1 [Reference]	1 [Reference]
31-50	0.62 (0.45-0.85)^b^	0.66 (0.48-0.91)^b^
51-64	0.52 (0.38-0.72)^b^	0.55 (0.47-0.91)^b^
≥65	0.44 (0.33-0.60)^b^	0.74 (0.48-1.14)
Sex		
Female	1 [Reference]	1 [Reference]
Male	1.32 (1.10-1.60)^b^	1.25 (1.02-1.54)^b^
Race and ethnicity		
American Indian and Alaska Native	0.80 (0.43-1.47)	0.83 (0.42-1.64)
Hispanic	1.36 (1.04-1.77)^b^	1.23 (0.93-1.62)
Non-Hispanic Asian	1.29 (0.72-2.31)	1.11 (0.58-2.12)
Non-Hispanic Black	1.28 (0.92-1.77)	1.51 (1.07-2.14)^b^
Non-Hispanic White	1 [Reference]	1 [Reference]
Other^c^	2.33 (0.93-5.84)	2.44 (1.03-5.79)^b^
Household income		
≥400% FPL	1 [Reference]	1 [Reference]
200%-399% FPL	0.98 (0.79-1.21)	1.12 (0.89-1.40)
100%-199% FPL	0.74 (0.58-0.94)	1.00 (0.74-1.34)
<100% FPL	0.90 (0.66-1.22)	1.40 (0.98-2.01)
US region		
Northeast	1 [Reference]	1 [Reference]
Midwest	1.03 (0.73-1.46)	1.14 (0.80-1.63)
South	0.96 (0.70-1.31)	0.96 (0.69-1.34)
West	0.90 (0.63-1.27)	0.84 (0.59-1.20)
Education		
Master’s or more	1 [reference]	1 [reference]
Some college	1.20 (0.90-1.59)	1.35 (1.00-1.82)
High school or less	1.00 (0.74-1.36)	1.21 (0.87-1.70)
Employment		
Yes	1 [Reference]	1 [Reference]
No	0.56 (0.46-0.67)^b^	0.67 (0.53-0.84)^b^
Insurance^d^		
Private only	1 [Reference]	1 [Reference]
Medicare	0.62 (0.47-0.82)^b^	0.94 (0.62-1.42)
Medicaid or other public	0.71 (0.55-0.93)^b^	0.85 (0.62-1.18)
Other coverage	0.59 (0.41-0.84)^b^	0.80 (0.54-1.17)
Uninsured	1.29 (0.91-1.82)	1.28 (0.85-1.91)
Smoking		
Never	1 [Reference]	1 [Reference]
Former	0.74 (0.60-0.91)^b^	0.89 (0.72-1.11)
Current	0.64 (0.48-0.85)^b^	0.80 (0.59-1.09)
Body mass index^e^		
Nonoverweight (<25.0)	1 [Reference]	1 [reference]
Overweight (25.0-29.9)	1.04 (0.82-1.31)	1.08 (0.84-1.38)
Obesity (≥30)	0.90 (0.72-1.13)	1.01 (0.79-1.28)
No. of COVID-19 vaccinations		
≥3	1 [Reference]	1 [reference]
1-2	1.14 (0.93-1.41)	0.99 (0.79-1.24)
0	0.89 (0.72-1.11)	0.77 (0.60-1.99)
No. of chronic conditions	0.75 (0.70-0.80)^b^	0.82 (0.75-0.89)^b^

Abbreviations: FPL, federal poverty level; OR, odds ratio.

^a^ In multiple logistic regression, adjusted ORs and 95% CIs were calculated for recovering from long COVID after adjusting for age, sex, race and ethnicity, household income, region, education, employment, insurance, smoking history, weight status, number of COVID-19 vaccinations, and other chronic conditions (arthritis, asthma, cancer, chronic obstructive pulmonary disease, diabetes, and hypertension), among the population that ever reported experiencing long COVID. Sample weights were applied to all analyses to account for nonresponse bias and produce nationally representative population estimates. For categorical variables, missing values were replaced with a distinct placeholder value (9999). For number of chronic conditions (ordinal variable) and COVID-19 vaccination status, missing values were dropped.

^b^ Statistical significance (*P* < .05).

^c^ Other included multiracial individuals and people whose race group was not releasable for respondent confidentiality.

^d^ Respondents classified as having Medicare included those who were dual eligible for Medicaid and Medicare Advantage patients.

^e^ Calculated as weight in kilograms divided by height in meters squared.

Subgroup analyses revealed notable differences in the association of food insecurity with current long COVID based on food assistance and employment status (Table 4). Food insecurity was not significantly associated with current long COVID among individuals receiving SNAP benefits or unemployed individuals. However, food insecurity was associated with current long COVID among those not receiving SNAP (aOR, 2.04; 95% CI, 1.58-2.64) and unemployed individuals (aOR, 2.32; 95% CI, 1.71-3.14). Food insecurity was significantly associated with current long COVID for people with household incomes at 200% FPL or less (aOR, 1.45; 95% CI, 1.09-1.93) and greater than 200% FPL (aOR, 2.37; 95% CI, 1.72-3.28). In multiple regression, SNAP participation (*P* for interaction = .04) and unemployment (*P* for interaction = .04) significantly modified the association of food insecurity with current long COVID (Figure).

**Table 4.** Adjusted Odds of Current Long COVID in Subgroup Analyses by Food Insecurity Status, SNAP Participation, and Employment Status

Characteristic by food insecurity status	Current long COVID, adjusted OR (95% CI)^a^
SNAP recipient^b^	
Yes (n = 1499)	
Food secure	1 [Reference]
Food insecure	1.23 (0.81-1.88)
No (n = 14 450)	
Food secure	1 [Reference]
Food insecure	2.04 (1.58-2.64)^c^
Employment	
Unemployed (n = 5796)^d^	
Food secure	1 [Reference]
Food insecure	1.28 (0.94-1.74)
Employed (n = 10 228)	
Food secure	1 [Reference]
Food insecure	2.32 (1.71-3.14)^c^
FPL	
≤200% (n = 4036)^e^	
Food secure	1 [Reference]
Food insecure	1.45 (1.09-1.93)^c^
>200% (n = 11 999)	
Food secure	1 [Reference]
Food insecure	2.37 (1.72-3.28)^c^

Abbreviations: FPL, federal poverty level; SNAP, Supplemental Nutrition Assistance Program.

^a^ Sample weights were applied to all analyses to account for nonresponse bias and produce nationally representative population estimates. For categorical variables, missing values were replaced with a distinct placeholder value (9999). For number of chronic conditions (ordinal variable) and COVID-19 vaccination status, missing values were dropped.

^b^ These multiple regression analyses were conducted in subpopulations of those with and without SNAP, adjusting for age, sex, race and ethnicity, household income, region, education, employment, insurance smoking history, weight status, number of COVID-19 vaccinations, and other chronic conditions (arthritis, asthma, cancer, chronic obstructive pulmonary disease, diabetes, and hypertension).

^c^ Statistical significance (*P* < .05).

^d^ These multiple regression analyses were conducted in subpopulations of those with and without employment, adjusting for age, sex, race and ethnicity, household income, region, education, insurance, smoking history, weight status, number of COVID-19 vaccinations, and other chronic conditions (arthritis, asthma, cancer, chronic obstructive pulmonary disease, diabetes, and hypertension).

^e^ These multiple regression analyses were conducted in subpopulations of those in households with FPL (≤200% or >200%), adjusting for age, sex, race and ethnicity, region, education, employment, insurance, smoking history, weight status, number of COVID-19 vaccinations, and other chronic conditions (arthritis, asthma, cancer, chronic obstructive pulmonary disease, diabetes, and hypertension).

**Figure.**
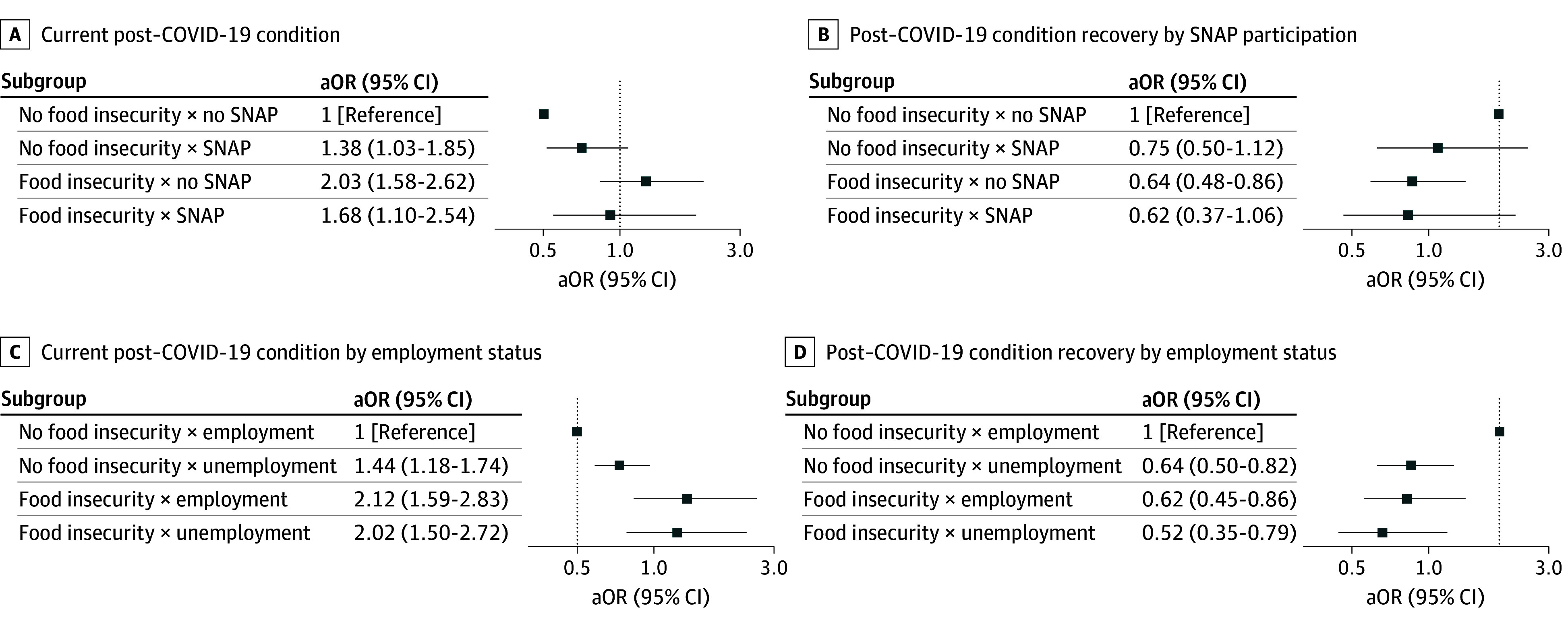
Adjusted Odds of Current Long COVID Accounting for Interactions Between Food Insecurity Status, Supplemental Nutrition Assistance Program (SNAP) Participation, and Employment Status These multiple regression analyses included interaction terms between food insecurity and SNAP participation or employment, adjusting for age, sex, race and ethnicity, household income, region, education, employment, insurance, smoking history, weight status, number of COVID-19 vaccinations, and other chronic conditions (arthritis, asthma, cancer, chronic obstructive pulmonary disease, diabetes, and hypertension). Sample weights were applied to all analyses to account for nonresponse bias and produce nationally representative population estimates. For categorical variables, missing values were replaced with a distinct placeholder value (9999). For number of chronic conditions (ordinal variable) and COVID-19 vaccination status, missing values were dropped. aOR indicates adjusted odds ratio.

In Poisson regression, food insecurity remained significantly associated with higher risk of current long COVID (adjusted relative risk, 1.60; 95% CI, 1.33-1.92) (eTable 1 in Supplement 1). In multiple logistic regression, food insecurity remained significantly associated with higher odds of current long COVID (aOR, 1.59; 95% CI, 1.33-1.90) when including individuals with unknown vaccination status (eTable 2 in Supplement 1). For long COVID recovery, food insecurity was associated with lower odds of recovery (aOR, 0.76; 95% CI, 0.60-0.96) (eTable 3 in Supplement 1). Notably, individuals with unknown vaccination status had higher odds of current long COVID (aOR, 1.89; 95% CI, 1.61-2.21) and lower odds of recovery compared with those with 3 or more vaccinations (aOR, 0.78; 95% CI, 0.63-0.97).

## Discussion

In this retrospective cross-sectional survey study, we analyzed data from surveys of US adults using the 2022 to 2023 NHIS and found that experiencing food insecurity was associated with increased odds of having current long COVID. Notably, we also found that participation in SNAP, the largest government-funded food assistance program in the US, attenuated the association of food insecurity with long COVID. Furthermore, our findings suggest that for adults who had long COVID, experiencing food insecurity was associated with a reduced likelihood of recovering from long COVID symptoms.

The association of food insecurity with long COVID may be multifactorial. Financial distress, incurred by unemployment, can lead people to delay or avoid medical care.^26,27,28^ Food insecurity can also lead to reduced consumption of essential nutrients for long-term physiological functioning.^29,30^ Such factors may contribute to and exacerbate long COVID, akin to how food insecurity impacts other chronic conditions.^10^ Similarly, long COVID may increase the risk of food insecurity by impairing individuals’ ability to work and generating financial hardship, suggesting a potentially bidirectional association.

Prior studies have demonstrated significant associations of unemployment with long COVID, unemployment with food insecurity, as well as unemployment benefits with reduced food insecurity.^4,13,24^ Our study found that food insecurity was associated with current long COVID in employed adults and for those not receiving SNAP. During the COVID-19 pandemic, federal expansion of SNAP benefits appeared to decrease the prevalence of food insecurity.^31^ This was followed by increases in food insecurity with subsequent reductions in SNAP benefits after the expiration of the COVID-19 emergency allotments.^32,33^ Additionally, the federal Coronavirus Aid, Relief, and Economic Security Act increased unemployment benefits, which has been associated with decreased food insecurity.^12,13^ Moreover, work requirements in SNAP led to decreased program participation.^34^

Food assistance programs, such as SNAP, offer an opportunity to mitigate the impact of food insecurity on individuals with long COVID. With the long-term costs of long COVID estimated to be $2.6 trillion, investing in SNAP represents a cost-effective strategy to reduce its burden and improve population health.^35,36^ We found that more than two-thirds of people with food insecurity did not participate in SNAP, in line with national estimates that suggest one-half of US adults with food insecurity do not participate in SNAP.^11^ In our study, most people who were food insecure and did not participate in SNAP were above 100% FPL; however, we also found 40% of people who were food insecure and below 100% FPL did not participate in SNAP, pointing to additional barriers such as the burdensome SNAP application process.^32^ Education about existing food assistance programs, expansion of food insecurity screenings, and greater federal and state investment in food assistance programs are needed to help close this gap.^37^

### Limitations

Our study has several key limitations. First, the NHIS dataset captures long COVID by self-report and may be subject to demographic variations in self-reporting. This limitation in some aspects mirrors the reality of diagnosing long COVID, which relies on self-reported symptoms beyond 3 months after diagnosis of COVID-19.^38^ Due to the cross-sectional nature of our study, we cannot infer causality or directionality, nor examine these associations over time. The NHIS survey questions do not capture precise timing for initial infection, long COVID onset, or resolution; and may be subject to recall bias. Many questions, however, were framed as lifetime occurrences (eg, ever diagnosed or ever had long COVID), reducing variability in recall across respondents. The long COVID recovery variable does not distinguish between those who recovered recently vs over a year ago, which may limit comparability in relation to food insecurity risk.

Due to sample size constraints, we analyzed a binary classification of food insecurity, which limited our ability to explore a possible dose-response association of a categorical food insecurity variable with long COVID. NHIS data had a relatively high nonresponse rate for COVID-19 vaccination status (26%); however, our results remained unchanged in sensitivity analyses that included those with unknown vaccination status. Additionally, NHIS data do not include variables that capture Paxlovid use, which could influence examined associations.

## Conclusions

To our knowledge, this is the first study that examines the association of food insecurity with long COVID utilizing a nationally representative dataset. In this survey study of US adults, food insecurity was associated with increased odds of having current long COVID and with a reduced likelihood of recovery among those affected. SNAP participation and unemployment may both attenuate the association of food insecurity with long COVID. Expanding SNAP eligibility, simplifying enrollment, and increasing awareness are critical to addressing food insecurity and its health impacts. Our findings underscore the importance of closing such gaps for long COVID and add to the mounting evidence for the role of food insecurity in chronic disease prevention and management.

## Notes

Supplement 1. **eTable 1.** Unadjusted and Adjusted Relative Risks of Current Long COVID Using Poisson Regression **eTable 2.** Unadjusted and Adjusted Odds Ratios of Current Long COVID by Respondent Characteristics, Including Unknown Vaccination Status **eTable 3.** Unadjusted and Adjusted Odds Ratios of Long COVID Recovery by Respondent Characteristics, Including Unknown Vaccination Status

Supplement 2.
Data Sharing Statement
