# Error in Key Points

**DOI:** 10.1001/jamanetworkopen.2025.55260

**Published:** 2026-01-02

In the Original Investigation titled “Long COVID and Food Insecurity in US Adults, 2022-2023,”^1^ published September 9, 2025, there was an error in the Key Points. It was reported that food insecurity was associated with a 78% higher chance of having current long COVID and a 34% lower chance of recovering from it; these percentages should have been 73% and 30%, respectively. This article has been corrected.^1^
