# Supplementary material for: Long COVID and Food Insecurity in US Adults, 2022-2023
Source: JAMA Netw Open. 2025 Sep 9;8(9):e2530730. doi: 10.1001/jamanetworkopen.2025.30730 (PMC12421344; doi:10.1001/jamanetworkopen.2025.30730)
Supplement: Supplement 1. — eTable 1. Unadjusted and Adjusted Relative Risks of Current Long COVID Using Poisson Regression eTable 2. Unadjusted and Adjusted Odds Ratios of Current Long COVID by Respondent Characteristics, Including Unknown Vaccination Status eTable 3. Unadjusted and Adjusted Odds Ratios of Long COVID Recovery by Respondent Characteristics, Including Unknown Vaccination Status [file jamanetwopen-e2530730-s001.pdf]

Supplemental Online Content

Lin JC, McCarthy M, Potluri S, Nguyen D, Yan R, Aysola J. Long COVID and food insecurity in US adults, 2022-2023. *JAMA Netw. Open*. 2025;8(9):e2530730.
doi:10.1001/jamanetworkopen.2025.30730

eTable 1. Unadjusted and Adjusted Odds Ratios of Long COVID Recovery by Respondent Characteristics, Including Unknown Vaccination Status
eTable 2. Unadjusted and Adjusted Relative Risks of Current Long COVID Using Poisson Regression
eTable 2. Unadjusted and Adjusted Odds Ratios of Current Long COVID by Respondent Characteristics, Including Unknown Vaccination Status

This supplemental material has been provided by the authors to give readers additional information about their work.

© 2025 Lin JC et al. *JAMA Network Open*.

eTable 1: Unadjusted and Adjusted Relative Risks of Current Long COVID Using Poisson Regression

Characteristic	Unadjusted Relative Risk (95% CI)	Adjusted Relative Risk[a] (95% CI)
**Food Insecurity Status**		
Food Security	1 [reference]	1 [reference]
Food Insecurity	**2.25 (1.92-2.65)***	**1.58 (1.32-1.90)***
**Age**		
18-30	1 [reference]	1 [reference]
31-50	**1.79 (1.40-2.28)***	**1.58 (1.24-2.01)***
51-64	**2.04 (1.61-2.57)***	**1.48 (1.15-1.90)***
65+	**1.89 (1.49-2.40)***	1.09 (0.80-1.50)
**Sex**		
Female	1 [reference]	1 [reference]
Male	**0.57 (0.50-0.65)***	**0.61 (0.53-0.70)***
**Race/Ethnicity**		
NH White	1 [reference]	1 [reference]
Hispanic	0.85 (0.69-1.05)	0.92 (0.75-1.12)
NH Black	0.79 (0.62-1.01)	**0.70 (0.55-0.89)***
NH Asian	**0.47 (0.30-0.74)***	0.66 (0.41-1.07)
AIAN	1.30 (0.89-1.92)	0.98 (0.66-1.46)
Other[b]	0.63 (0.30-1.34)	0.71 (0.35-1.45)
**Household Income**		
<100% FPL	1 [reference]	1 [reference]
100-199% FPL	**1.29 (1.09-1.52)***	1.05 (0.89-1.25)
200-399% FPL	**1.72 (1.43-2.06)***	1.11 (0.90-1.37)
400+% FPL	**1.72 (1.39-2.13)***	0.93 (0.71-1.20)

© 2025 Lin JC et al. *JAMA Network Open*.

**Education**		
High school or less	1 [reference]	1 [reference]
Some college	**1.36 (1.11-1.66)***	1.04 (0.85-1.28)
Master's or more	**1.47 (1.18-1.83)***	0.93 (0.73-1.19)
**Employment**		
Yes	1 [reference]	1 [reference]
No	**1.72 (1.52-1.96)***	**1.29 (1.09-1.51)***
**Health Insurance**		
Private	1 [reference]	1 [reference]
Medicare[c]	**1.41 (1.17-1.69)***	1.06 (0.82-1.36)
Medicaid and other public	**1.76 (1.48-2.11)***	1.15 (0.93-1.42)
Other coverage	**1.78 (1.41-2.25)***	1.22 (0.95-1.56)
Uninsured	1.04 (0.79-1.36)	0.94 (0.70-1.25)
**US Region, n (%)**		
Northeast	1 [reference]	1 [reference]
Midwest	1.26 (1.00-1.59)	1.08 (0.86-1.37)
South	**1.32 (1.06-1.64)***	1.16 (0.93-1.45)
West	**1.35 (1.06-1.73)***	**1.35 (1.07-1.71)***
**Smoking**		
Never	1 [reference]	1 [reference]
Former	**1.51 (1.30-1.75)***	**1.20 (1.03-1.40)***
Current	**1.82 (1.50-2.20)***	1.23 (1.00-1.51)
**Body Mass Index**		
Non-Overweight	1 [reference]	1 [reference]
Overweight	1.11 (0.94-1.32)	1.05 (0.88-1.24)
Obesity	**1.58 (1.34-1.87)***	**1.21 (1.02-1.43)***

© 2025 Lin JC et al. *JAMA Network Open*.

No. COVID-19 vaccinations		
3+	1 [reference]	1 [reference]
1-2	1.12 (0.97-1.31)	**1.22 (1.04-1.43)***
0	**1.61 (1.38-1.88)***	**1.64 (1.38-1.95)***
**No. chronic conditions**	**1.46 (1.40-1.52)***	**1.35 (1.28-1.43)***

AIAN, American Indian/Alaska Native; CI, Confidence Interval; FPL, federal poverty level; NH, Non-Hispanic. Statistically significant relationships are bolded.

*Asterisks denoted statistical significance (p<0.05).

[a]In Poisson regression, we calculated adjusted odds ratios and 95% confidence intervals for having current long COVID after adjusting for age, sex, race and ethnicity, household income, region, education, smoking history, insurance, employment, weight status, number of COVID-19 vaccinations, and other chronic conditions (arthritis, asthma, cancer, chronic obstructive pulmonary disease, diabetes, hypertension), among the population that reported experiencing COVID. We applied sample weights to all analyses to account for non-response bias and produce nationally representative population estimates. For categorical variables, we replaced missing values with a distinct placeholder value (9999). For number of chronic conditions (ordinal variable), we dropped missing values.
[b]Other included multiracial individuals and people whose race group was not releasable for respondent confidentiality.
[c]Respondents classified as having Medicare included those who were dual eligible for Medicaid and Medicare Advantage patients.

© 2025 Lin JC et al. *JAMA Network Open*.

eTable 2: Unadjusted and Adjusted Odds Ratios of Current Long COVID by Respondent Characteristics, Including Unknown Vaccination Status

**Characteristics (N=21,624)**	**Unadjusted OR (95% CI)[a]**	**Adjusted OR (95% CI)[a]**
Food Insecurity Status		
Food Secure	1 [reference]	1 [reference]
Food Insecure	**2.21 (1.88-2.59)***	**1.59 (1.33-1.90)**
Age		
18-30	1 [reference]	1 [reference]
31-50	**1.98 (1.61-2.43)***	**1.76 (1.43-2.16)***
51-64	**2.17 (1.77-2.66)***	**1.51 (1.21-1.88)***
65+	**2.04 (1.67-2.49)***	1.05 (0.79-1.38)
Sex		
Female	1 [reference]	1 [reference]
Male	**0.56 (0.50-0.63)***	0.58 (0.51-0.66)
Race/Ethnicity		
NH White	1 [reference]	1 [reference]
Hispanic	0.88 (0.73-1.05)	0.88 (0.73-1.07)
NH Black	**0.80 (0.66-0.98)***	**0.65 (0.53-0.81)***
NH Asian	**0.44 (0.31-0.64)***	**0.58 (0.40-0.85)***
AIAN	1.38 (0.94-2.02)	1.03 (0.67-1.58)
Other[b]	0.75 (0.41-1.36)	0.78 (0.42-1.43)
Household Income		
400+% FPL	1 [reference]	1 [reference]
200-399% FPL	**1.32 (1.14-1.52)***	1.03 (0.80-1.32)
100-199% FPL	**1.70 (1.45-2.00)***	**1.23 (1.01-1.49)***
<100% FPL	**1.67 (1.36-2.04)***	1.03 (0.98-1.34)
US Region		
Northeast	1 [reference]	1 [reference]
Midwest	**1.24 (1.01-1.51)***	1.09 (0.77-1.34)
South	**1.31 (1.09-1.58)***	1.20 (0.99-1.45)
West	**1.31 (1.06-1.61)***	**1.33 (1.09-1.63)***
Education		
Master's or more	1 [reference]	1 [reference]
Some college	**1.25 (1.06-1.47)***	1.02 (0.86-1.21)
High school or less	**1.35 (1.13-1.62)***	0.94 (0.77-1.15)
Employment		
Yes	1 [reference]	1 [reference]
No	**1.62 (1.44-1.81)***	**1.22 (1.05-1.41)***

© 2025 Lin JC et al. *JAMA Network Open*.

Insurance[c]		
Private only	1 [reference]	1 [reference]
Medicare	**1.39 (1.18-1.63)***	1.10 (0.87-1.39)
Medicaid or other public	**1.58 (1.33-1.87)***	1.06 (0.85-1.30)
Other coverage	**1.83 (1.47-2.29)***	**1.33 (1.04-1.69)***
Uninsured	0.97 (0.76-1.24)	0.90 (0.68-1.18)
Smoking		
Never	1 [reference]	1 [reference]
Former	**1.48 (1.30-1.69)***	**1.21 (1.05-1.39)***
Current	**1.68 (1.41-2.00)***	1.14 (0.94-1.38)
Body Mass Index		
Non-Overweight	1 [reference]	1 [reference]
Overweight	1.12 (0.96-1.29)	1.04 (0.89-1.22)
Obesity	**1.66 (1.43-1.92)***	**1.21 (1.03-1.42)***
No. COVID-19 vaccinations		
3+	1 [reference]	1 [reference]
1-2	1.13 (0.96-1.33)	**1.22 (1.03-1.45)***
0	**1.67 (1.41-1.98)***	**1.71 (1.42-2.06)***
Unknown	**1.82 (1.41-1.98)***	**1.89 (1.61-2.21)***
No. chronic conditions	**1.49 (1.43-1.56)***	**1.40 (1.32-1.49)***

AIAN, American Indian/Alaska Native; CI, Confidence Interval; FPL, federal poverty level; NH, Non-Hispanic; OR, Odds Ratio; SE, standard error. Statistically significant relationships are bolded.

*Asterisks denoted statistical significance (p<0.05).
[a]In multiple logistic regression, we calculated adjusted odds ratios and 95% confidence intervals for having current long COVID after adjusting for age, sex, race and ethnicity, household income, region, education, employment, insurance, smoking history, weight status, number of COVID-19 vaccinations, and other chronic conditions (arthritis, asthma, cancer, chronic obstructive pulmonary disease, diabetes, hypertension), among the population that reported experiencing COVID-19, including people with unknown vaccination status. We applied sample weights to all analyses to account for non-response bias and produce nationally representative population estimates. For categorical variables, we replaced missing values with a distinct placeholder value (9999). For number of chronic conditions (ordinal variable), we dropped missing values.
[b]Other included multiracial individuals and people whose race group was not releasable for respondent confidentiality.
[c]Respondents classified as having Medicare included those who were dual eligible for Medicaid and Medicare Advantage patients.

© 2025 Lin JC et al. *JAMA Network Open*.

© 2025 Lin JC et al. *JAMA Network Open*.

eTable 3: Unadjusted and Adjusted Odds Ratios of Long COVID Recovery by Respondent Characteristics, Including Unknown Vaccination Status

Characteristics (N=3876)	Unadjusted OR (95% CI)	Adjusted OR (95% CI)[a]
Food Insecurity Status		
Food Secure	1 [reference]	1 [reference]
Food Insecure	**0.75 (0.61-0.93)***	**0.76 (0.60-0.96)**
Age		
18-30	1 [reference]	1 [reference]
31-50	**0.57 (0.45-0.73)***	**0.61 (0.45-0.75)***
51-64	**0.53 (0.41-0.68)***	**0.68 (0.43-0.73)***
65+	**0.43 (0.34-0.55)***	0.76 (0.53-1.08)
Sex		
Female	1 [reference]	1 [reference]
Male	**1.31 (1.12-1.54)***	**1.28 (1.09-1.52)**
Race/Ethnicity		
NH White	1 [reference]	1 [reference]
Hispanic	**1.38 (1.11-1.72)***	**1.26 (1.00-1.59)***
NH Black	1.23 (0.95- 1.60)	**1.46 (1.10-1.92)***
NH Asian	1.30 (0.82- 2.06)	1.19 (0.73-1.97)
AIAN	1.07 (0.56- 2.01)	1.09 (0.56-2.14)
Other[b]	1.91 (0.95-3.84)	1.81 (0.90-3.63)
Household Income		
400+% FPL	1 [reference]	1 [reference]
200-399% FPL	0.98 (0.81-1.17)	1.20 (0.87-1.65)
100-199% FPL	**0.78 (0.63-0.97)***	0.94 (0.73-1.21)
<100% FPL	0.93 (0.72-1.20)	1.04 (0.86-1.26)
US Region		
Northeast	1 [reference]	1 [reference]
Midwest	0.94 (0.72-1.23)	0.99 (0.75-1.31)
South	0.84 (0.65-1.08)	0.82 (0.63-1.07)
West	0.91 (0.69-1.20)	0.83 (0.62-1.11)
Education		
Master's or more	1 [reference]	1 [reference]
Some college	**1.32 (1.05-1.66)***	**1.37 (1.08-1.72)***
High school or less	1.11 (0.87- 1.42)	1.17 (0.89-1.53)
Employment		
Yes	1 [reference]	1 [reference]
No	**0.64 (0.55-0.74)***	**0.77 (0.64-0.93)***

© 2025 Lin JC et al. *JAMA Network Open*.

Insurance[c]		
Private only	1 [reference]	1 [reference]
Medicare	**0.64 (0.51-0.80)***	0.85 (0.61-1.17)
Medicaid or other public	0.88 (0.70-1.11)	0.99 (0.75-1.31)
Other coverage	**0.63 (0.46-0.87)***	0.80 (0.58-1.11)
Uninsured	**1.41 (1.05-1.90)***	1.36 (0.96-1.91)
Smoking		
Never	1 [reference]	1 [reference]
Former	**0.76 (0.64-0.91)***	0.88 (0.73-1.06)
Current	**0.71 (0.55-0.91)***	0.89 (0.68-1.16)
Body Mass Index		
Non-Overweight	1 [reference]	1 [reference]
Overweight	1.03 (0.85-1.25)	1.10 (0.90-1.35)
Obesity	0.95 (0.79- 1.15)	1.12 (0.92-1.37)
No. COVID-19 vaccinations		
3+	1 [reference]	1 [reference]
1-2	1.14 (0.93-1.41)	1.04 (0.83-1.29)
0	0.90 (0.72-1.11)	0.80 (0.63-1.02)
Unknown	0.87 (0.71-1.08)	**0.78 (0.63-0.97)***
No. chronic conditions	**0.77 (0.73-0.82)***	**0.83 (0.77-0.90)***

AIAN, American Indian/Alaska Native; CI, Confidence Interval; FPL, federal poverty level; NH, Non-Hispanic; OR, Odds Ratio; SE, standard error. Statistically significant relationships are bolded.

*Asterisks denoted statistical significance (p<0.05).

[a]In multiple logistic regression, we calculated adjusted odds ratios and 95% confidence intervals for recovering from long COVID after adjusting for age, sex, race and ethnicity, household income, region, education, smoking history, insurance, employment, weight status, number of COVID-19 vaccinations, and other chronic conditions (arthritis, asthma, cancer, chronic obstructive pulmonary disease, diabetes, hypertension), among the population that reported experiencing long COVID, including people with unknown vaccination status. We applied sample weights to all analyses to account for non-response bias and produce nationally representative population estimates. For categorical variables, we replaced missing values with a distinct placeholder value (9999). For number of chronic conditions (ordinal variable), we dropped missing values.

[b]Other included multiracial individuals and people whose race group was not releasable for respondent confidentiality.

© 2025 Lin JC et al. *JAMA Network Open*.

[c]Respondents classified as having Medicare included those who were dual eligible for Medicaid and Medicare Advantage patients.

© 2025 Lin JC et al. *JAMA Network Open*.
