# Supplementary material for: Long COVID and Food Insecurity in US Adults, 2022-2023
Source: JAMA Netw Open. 2025 Sep 9;8(9):e2530730. doi: 10.1001/jamanetworkopen.2025.30730 (PMC12421344; doi:10.1001/jamanetworkopen.2025.30730)
Supplement: Supplement 2. — Data Sharing Statement [file jamanetwopen-e2530730-s002.pdf]

Data Sharing Statement

Lin. Long COVID and Food Insecurity in US Adults, 2022-2023. *JAMA Netw Open*. Published September 09, 2025. doi:10.1001/jamanetworkopen.2025.30730

Data
Data available: Yes
Data types: Data (not involving human participants)
How to access data: All data and documentation are available online at the CDC website: http://cdc.gov/nchs/nhis/documentation/
When available: With publication

Supporting Documents
Document types: Other (please specify)
Additional Information: All data and documentation are available online at the CDC website: http://cdc.gov/nchs/nhis/documentation/
How to access documents: All data and documentation are available online at the CDC website: http://cdc.gov/nchs/nhis/documentation/
When available: With publication

Additional Information
Who can access the data: All data and documentation are available online at the CDC website to all interested parties for any purpose: http://cdc.gov/nchs/nhis/documentation/
Types of analyses: All data and documentation are available online at the CDC website to all interested parties for any purpose: http://cdc.gov/nchs/nhis/documentation/
Mechanisms of data availability: All data and documentation are available online at the CDC website to all interested parties for any purpose: http://cdc.gov/nchs/nhis/documentation/
